# High-Quality Genome Sequences of Six Actinobacterial Strains Isolated from Granite, Granodiorite, and Tourmaline Rock Surfaces Sampled from Tamil Nadu, India, and New England, United States

**DOI:** 10.1128/mra.00946-22

**Published:** 2022-10-26

**Authors:** Abdellatif Gueddou, Nathaniel J. Ennis, Dhanasekaran Dharumadurai, Louis S. Tisa

**Affiliations:** a Department of Molecular, Cellular and Biomedical Sciences, University of New Hampshire, Durham, New Hampshire, USA; b Department of Microbiology, Bharathidasan University, Tiruchirappalli, Tamil Nadu, India; University of Southern California

## Abstract

Here, we announce four contiguous and two high-quality draft genome sequences of six actinobacterial strains (*Blastococcus, Georgenia, Nocardioides, Allobranchiibius, Yimella,* and *Williamsia*) that were isolated from rock samples obtained from Indian historical ruins and colonial building stones in New England, United States. These new sequences expand the genome datasets recovered from stone-dwelling microbes and will allow the prediction of their potential role in the stone microbiome.

## ANNOUNCEMENT

Using culture-independent and -dependent approaches, we have been studying the community of the stone microbiome of different stone lithologies under various environmental conditions (1–5). Previously, we described 17 draft genome sequences for six actinobacterial strains (1) and 11 members of the stone-dwelling actinobacteria *Geodermatophilaceae* family (5). A total of 74 bacterial strains were isolated as described previously (1, 5) from stone surfaces acquired from New England and Indian ruins (1, 4, 5). These strains were isolated on Czapek (6), R2A (7), Luedemann (8), or starch casein (6) media, purified and stored at 80°C. Six of these bacterial strains (Table 1) were isolated from granite, granodiorite, and tourmaline rock surfaces and were chosen for whole-genome sequencing analysis to expand the genome datasets and help understand the rock microbiome function.

The bacterial cultures were revived from the −80°C stocks by streaking on Czapek agar plates. The revived cultures were incubated in Czapek broth medium for 5 days at 28°C. The cultures were collected by centrifugation at 14,000 g for 5 min. The cell pellet was homogenized by successive passing through a 0.1-mm sterile syringe needle. Genomic DNA (gDNA) was extracted by the cetyltrimethylammonium bromide (CTAB) DNA extraction method (9). RNA was removed by RNase treatment. The quality and quantity of the gDNA were verified using a Thermo Scientific Nanodrop 2000c spectrophotometer.

DNA libraries were prepared for long-read sequencing using the Rapid sequencing gDNA-barcoding kit (SQK-RBK004; Oxford Nanopore Technologies [ONT], Inc., United Kingdom) and loaded without size selection onto the primed MinION SpotON flow cell (R9.4.1 FLO-MIN 106; ONT) according to the manufacturer’s protocol. Whole-genome sequencing was conducted using an Oxford Nanopore MinION Mk1C device connected to MinKNOW version 22.05.8 for 48-h real-time base calling in high-accuracy mode using Guppy v6.1.5, with a quality score cut-off 7. For *de novo* assembly of the genomes, all quality reads that passed quality control in the FASTQ format were assembled using the EPI2ME Labs platform (ONT) employing the assembler Flye version 2.8.1-b1676 (10). The resulting assembles were polished using Racon version v1.4.16 (11), followed by a second polishing step using Medaka version v1.5.0 (12). Statistics of the final polished consensus sequences were calculated using QUAST v5.2.0 (13). The completeness and contamination of the assembled genomes were estimated by CheckM (14). Circularization of the genome was determined by the assembler program Flye version 2.8.1-b1676 (10) and confirmed by Bandage version.0.8.0 (15). The final polished genomes were annotated at NCBI with the Prokaryotic Genome Annotation Pipeline (PGAP) (16). All software was run using default parameters. The assembly metrics and annotation features are also given in Table 1. Four genomes were singletons and two were drafts with less than six contigs. *Georgenia* sp. strain TF02-10 had three plasmids.

The strains were identified by a whole-genome-based taxonomic analysis the Type Strain Genome Server (TYGS), bioinformatics platform (11) available at https://tygs.dsmz.de. Based on digital DNA:DNA hybridization (dDDH) values less than 70 % (17) and average nucleotide identity (ANI) values less than 95 % (18), *Blastococcus* PFR04-17, *Georgenia* TF02-10, and *Nocardiodes* TF02-7 represent potential new species. Isolates NH-cas1, DF01-3, and GilTou73 belong to Yimella lutea, Williamsia marianensis, and Allobranchiibius huperziae, respectively.

### Data availability.

The genome sequences of these bacterial strains have been deposited in GenBank under the accession numbers listed in Table 1. Both the assembly and raw reads are available at DDBJ/ENA/GenBank under BioProject number PRJNA694661.

**TABLE 1:** Genome statistics

Bacterial species	Isolate^*a*^	Accession no.	SRA^*b*^ accession no.	No. of reads	No. of contigs	Avg coverage (X)	CheckM^*c*^ completion (contamination)	Circular genome^*d*^	Genome size (bp)	No. of plasmids	ANI value^*e*^ best hit (accession no.)	G+C content (%)	No. of CDSs^*f*^
*Blastococcus* sp.	PFR04-17	CP095412	SRR18732977	1,362,941	1	471. 15	97.35 % (0.32 %)	Yes	4,585,415	-	84.3 % B. colisei (VFQE00000000)	73.0	4,599
*Georgenia* sp.	TF02-10	CP094289-CP094292	SRR18059838	58,591	1	48.79	99.18 % (0.87 %)	Yes	3,953,529	3	84.2% G. thermotolerans (WHJE00000000)	74.4	3,668
*Nocardioides* sp.	TF02-7	CP092535	SRR18059805	28,560	1	23.21	87.49 % (0.0 %)	Yes	4,745,229	-	% 83.6 % N. pelophilus (JACEHF000000000)	73.1	4,872
*Allobranchiibius* sp.	GilTou73	CP090051	SRR17266971	284,933	1	288.81	98.66 % (0.54 %)	Yes	3,552,513	-	97.5 % A. huperziae (JACCFW000000000)	69.63	3,432
*Yimella* sp.	NH-cas1	JAKSGG000000000	SRR18059840	105,543	3	104.09	99.24 % (0.72 %)	No	3,201,546	-	98.5% Y. lutea (VFMO00000000)	66.9	3,034
*Williamsia* sp.	DF01-3	JALKFS000000000	SRR18733175	1,067,309	6	442.9	97.94 % (0.36 %)	No	5,592,969	-	95.9 % W. marianensis (QEOM00000000)	64.6	5,252

a Bacteria were isolated on Czapek (PRF04-17, TF02-10, TF02-7) and R2A (GilTou73, NH-Cas1, DF01-3) media as described previously (1, 5). All bacterial strains were grown for 5 days before gDNA extraction. Stone sample location and lithology are listed as follows: PFR04-17, granodiorite from the upper damaged area of the temple at Fort Ranjankudi, in Perambalur, Tamil Nadu, India; TF02-7 and TF02-10, granite from outside rock damage area at Fort Tiruchirappalli, Tamil Nadu, India; GilTou73, tourmaline stone found in the park grounds at Gillette Castle State Park in East Haddam, CT, USA; NH-Cas1, granite from stone wall remains of the Lignold Casket Co. in Holderness, NH, USA; and DF01-3, granite from the outer part of the temple wall at Fort Dindigul in Dindigul, Tamil Nadu, India.

b Sequence Reads Archive.

c Genome assembly completeness and contamination were estimated by CheckM (14).

d Circularization of the genome was determined by the assembler program Flye version 2.8.1-b1676 (10) and confirmed by Bandage version.0.8.0 (15).

e ANI, average nucleotide identity. Values for the best hit and accession number. Values < 95% represent potential new species.

f CDSs, coding DNA sequences.
